# Identification of 5-Methoxy-2-(Diformylmethylidene)-3,3-Dimethylindole as an Anti-Influenza A Virus Agent

**DOI:** 10.1371/journal.pone.0170352

**Published:** 2017-01-23

**Authors:** Ming Cheang Tan, Wan Ying Wong, Wei Lun Ng, Kok Siong Yeo, Taznim Begam Mohd Mohidin, Yat-Yuen Lim, Fadhil Lafta, Hapipah Mohd Ali, Chee-Kwee Ea

**Affiliations:** 1 Institute of Biological Sciences, Faculty of Science, University of Malaya, Kuala Lumpur, Malaysia; 2 Department of Chemistry, Faculty of Science, University of Malaya, Kuala Lumpur, Malaysia; University of North Carolina at Chapel Hill School of Dentistry, UNITED STATES

## Abstract

Influenza virus is estimated to cause 3–5 million severe complications and about 250–500 thousand deaths per year. Different kinds of anti-influenza virus drugs have been developed. However, the emergence of drug resistant strains has presented a big challenge for efficient antiviral therapy. Indole derivatives have been shown to exhibit both antiviral and anti-inflammatory activities. In this study, we adopted a cell-based system to screen for potential anti-IAV agents. Four indole derivatives (named 525A, 526A, 527A and 528A) were subjected to the antiviral screening, of which 526A was selected for further investigation. We reported that pre-treating cells with 526A protects cells from IAV infection. Furthermore, 526A inhibits IAV replication by inhibiting the expression of IAV genes. Interestingly, 526A suppresses the activation of IRF3 and STAT1 in host cells and thus represses the production of type I interferon response and cytokines in IAV-infected cells. Importantly, 526A also partially blocks the activation of RIG-I pathway. Taken together, these results suggest that 526A may be a potential anti-influenza A virus agent.

## Introduction

Influenza is a respiratory illness that primarily infects human and birds. In tropical regions, the impact of influenza outbreak can be long-lasting, which leads to long-term economic burden and productivity loss. The world experienced four influenza pandemics which were the Spanish flu (1919), the Asian flu (1957), the Hong Kong flu (1968) and the H1N1 flu (2009). Annually, 5–10% of adults and 20–30% of children are vulnerable to influenza attacks according to World Health Organization. Worldwide, influenza is estimated to cause 3 to 5 million severe complications and about 250 000 to 500 000 deaths every year.

There are three types of influenza viruses–A, B, and C. Among them, type A influenza virus draws the most attention as it is highly infectious and the most virulent. Influenza A is a member of *Orthomyxoviridae* family. It is a negative sense, single-stranded, and segmented ribonucleic acid (RNA) virus[1]. It contains eight segments which encode for at least 11 viral genes such as hemagglutinin (HA), neuraminidase (NA), matrix protein 1 (M1), matrix protein 2 (M2), nucleoprotein (NP), non-structural protein 1 (NSP1), non-structural protein 2 (NSP2; also known as nuclear export protein, NEP), polymerase acidic protein (PA), polymerase basic protein 1 (PB1), polymerase basic protein 2 (PB2), and polymerase basic protein 1-F2 (PB1-F2)[2]. Influenza A is an enveloped virus as its outer layer is a lipid bilayer membrane which is taken from the host cell. This lipid bilayer carries three viral transmembrane proteins: HA, NA, and M2. Underneath the membrane is a layer of M1 and the core viral ribonucleoprotein (vRNP) complexes. VRNP consists of viral RNAs, heterotrimeric polymerase complex and nucleoprotein.

When influenza A virus (IAV) encounters a normal host cell, the binding of viral surface glycoprotein HA to the host cell-surface sialic acid receptors triggers the endocytosis of the virus. The influx of protons in the endosomes via M2 ion channel creates a low pH condition which further leads to a structural change in the HA protein and activates its role in the membrane fusion process[1]. Thereby, the vRNPs are released into the cytoplasm and transported into the nucleus. In the nucleus, the negative-sense strands of RNA will be converted into positive-sense strands of RNA to serve as templates for viral RNAs (vRNAs) production. The viral RNA polymerases play their role in “cap-snatching” mechanism to synthesize viral mRNAs with 5’ methylated capped RNA fragments cleaved from host pre-mRNAs[3]. From there, the viral mRNAs will be transported to the cytoplasm to produce viral proteins such as HA, M2 and NA. As an enveloped virus, it uses host cell’s plasma membrane to form viral particles and leaves the cell by cleaving sialic acids with the newly synthesized NA[1].

Indole is an aromatic heterocyclic organic compound with a bicyclic structure consisting of a six-membered ring fused to a five-membered nitrogen-containing pyrrole ring. Indoles are the most ubiquitous component of biologically active natural products and possess a wide range of biological activities[4]. With the unique characteristic of being able to mimic the structure of peptides and bind reversibly to enzymes[5], indole derivatives remain to be a fascinated subject to be studied in both academia and industry. Studies have found that indole derivatives exhibit anti-inflammatory[6], anti-microbial[7], and antiviral activities against several types of virus including Human Immunodeficiency Virus (HIV)[5], Herpes Simplex Virus (HSV) types 1, -2, Flavivirus, Respiratory Syncytial Virus (RSV)[8], and Coxsackie B virus[9]. In addition, some indole derivatives also protect red blood cells and DNA against radical-induced oxidation[10].

Antiviral drugs play a vital role in fast-spreading epidemics such as influenza A. Different kinds of anti-influenza virus drugs have been developed. However, the rise of drug resistance strains has become a big challenge for efficient antiviral therapy. We have previously described a cell-based screening system for identifying inhibitors of IAV replication[11]. In this study, we used this screening system to test four novel synthetic indole derivative agents (named in short 525A, 526A, 527A and 528A) for their anti-influenza A virus activities. We have shown that the indole derivative 526A is the most promising compound and was selected for further testing of its biological activity. Our results demonstrate that pre-treating cells with 526A protects cells from IAV infection. Furthermore, 526A inhibits IAV replication by mainly inhibiting the expression of IAV genes and proteins. Interestingly, 526A also suppresses the activation of IRF3 and STAT1 in host cells and thus represses the production of type I interferon response and cytokines in IAV-infected cells. 526A also inhibits the activation of RIG-I pathway. Taken together, these results suggest that 526A may be a potential anti-influenza A virus agent.

## Results

### Screening of four novel synthetic indole derivatives for antiviral property

**Fig 1:**
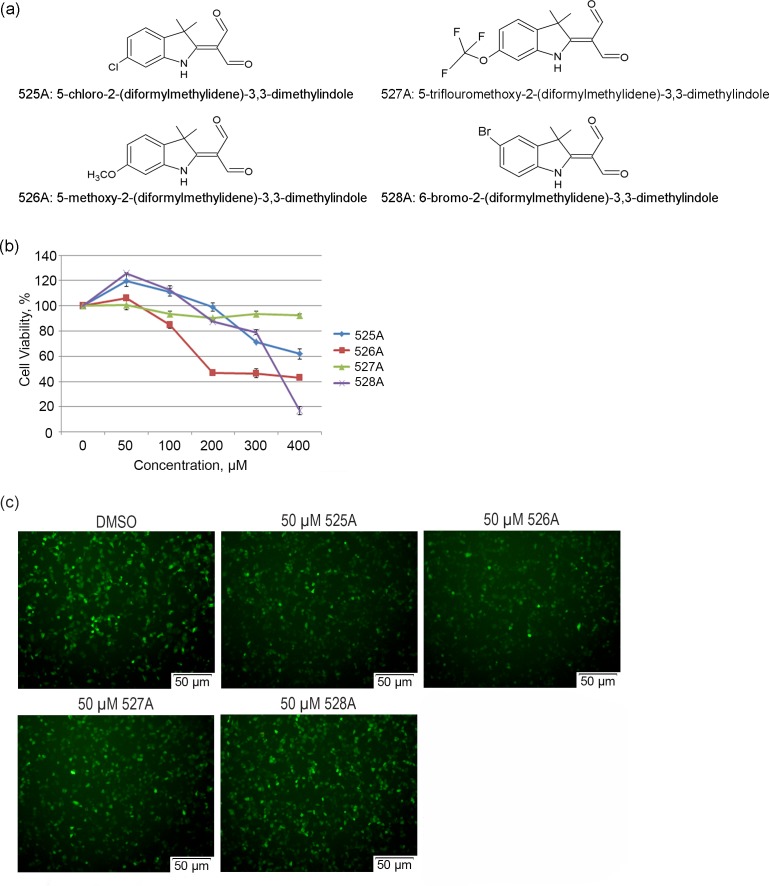
Screening of the four novel synthetic indole derivatives for antiviral property. (a) Chemical structures and chemical names of 525A, 526A, 527A and 528A. (b) A549-PB1 cells were seeded in a 96-well plate and pre-treated with various concentrations of the four compounds (525A, 526A, 527A and 528A) for two days. Cells viability was measured with a MTT assay. Error bars represent the variation range of triplicate experiments. (c) A549-PB1 cells were seeded in a 96-well plate and pre-treated with the four compounds at 50 μM for four hours followed by PR8-PB1flank-eGFP IAV infection at an MOI of 1.0 for eighteen hours. Fluorescence microscopy images were taken using an Olympus IX73 inverted microscope at 200x final magnification and photographed using an Olympus DP73 digital camera with Cellsens standard software.

Four new synthetic indole derivatives (Fig 1A) were tested for antiviral activity using a cell-based screening system for IAV replication inhibitors[11]. A non-replicative PR8 strain IAV carrying eGFP in place of the PB1 gene (PR8-PB1flank-eGFP) was used to infect A549 cells that stably express PB1 protein (A549-PB1). Upon PR8-PB1flank-eGFP viral infection and replication, infected A549-PB1 cells will express the eGFP reporter. Prior to studying the four indole derivatives on IAV infection, we determined the cytotoxic effect of each compound in A549-PB1 cells using a MTT assay. After two days of incubation, all compounds were found to be non-toxic at the concentration below 100 μM (Fig 1B) with the IC_50_ of 442 μM for 525A, 266 μM for 526A and 341 μM for 528A (Fig 1B and S1 Fig).

To screen if any of the indole derivatives possesses antiviral property, we pre-treated the A549-PB1 cells with 50 μM of the indole derivatives for four hours and infected the cells with PR8-PB1flank-eGFP IAV at a multiplicity of infection (MOI) of 1.0. We found no obvious reduction of eGFP-positive cells for both 527A and 528A-treated cells, and slightly less eGFP-positive cells in 525A-treated cells (Fig 1C). Interestingly, 526A pretreatment led to a moderate reduction of eGFP-positive cells (Fig 1C). The antiviral activity of 526A was not due to their cytotoxic effect as cell proliferation was unaffected at 50 μM (Fig 1B) and treated cells displayed normal morphology (S2 Fig). Thus, 526A was selected for further testing.

To determine if all four indole derivatives also exert antiviral activity against other viruses, we pre-treated A549-PB1 cells with each derivative for four hours and infected the cells with a vesicular stomatitis virus carrying a GFP (VSV-GFP) at an MOI of 1.0. More than 90% of GFP-positive cells were observed in DMSO-treated A549-PB1 cells while four compounds possess different levels reduction of GFP-positive cells (S3A and S3B Fig). Among the four, 525A showed the highest reduction of GFP-positive cells (S3A Fig). These results suggest that all four indole derivatives protect cells from VSV-GFP infection to varying degrees.

### 526A suppresses IAV replication

**Fig 2:**
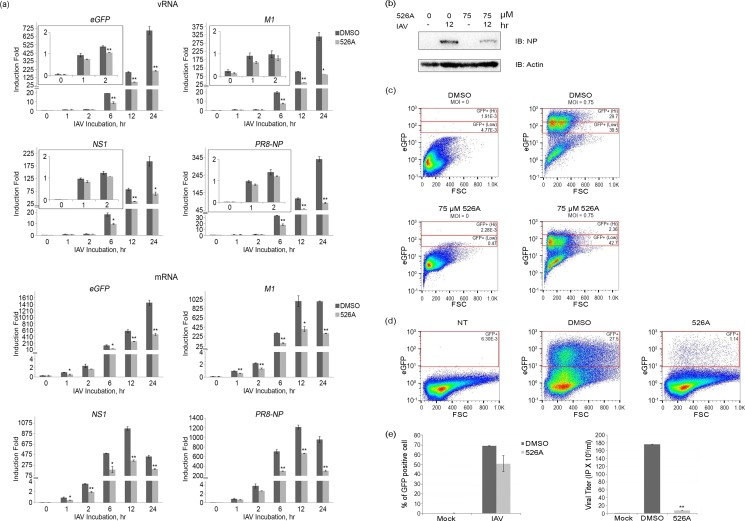
526A suppresses IAV replication. (a) A549-PB1 cells were pre-treated with or without 75 μM 526A for four hours and subjected to PR8-PB1flank-eGFP IAV infection at an MOI of 1.0 for the indicated time points. The expression of M1, NS1, PR8-NP and eGFP from both IAV negative-strand vRNA and positive-strand mRNA was measured with RT-qPCR (Table 1). Error bars represent the variation range of duplicate experiments. (b) A549-PB1 cells were treated with or without 75 μM 526A for four hours and subjected to PR8-PB1flank-eGFP IAV infection at an MOI of 1.0 for twelve hours. The whole cell extracts were collected and subjected to immunoblotting with antibodies against NP and actin. (c) A549-PB1 cells were pre-treated with or without 75 μM 526A for four hours and subjected to PR8-PB1flank-eGFP IAV infection at an MOI of 0.75 for eighteen hours. Cells were washed and fixed with 0.1% formaldehyde before titering with FACS. (d) A549-PB1 cells were infected with the harvested supernatant in Fig 2c and subjected to a viral titering assay using FACS. NT: non-treated sample. (e) The left panel shows the percentage of GFP-positive cells upon primary IAV infection in A549-PB1 cells while the right panel shows the viral titering of secondary IAV infection from the harvested viral supernatant. Error bars represent the variation range of duplicate experiments. IP: infectious viral particle. Student’s t-test: *, p < 0.05; **, p < 0.01.

To investigate whether 526A affects the replication of IAV, we studied the IAV polymerase activity by measuring the expression of both negative strand vRNAs and positive strand viral mRNA using RT-qPCR. A549-PB1 cells were pre-treated with 75 μM of 526A for four hours followed by PR8-PB1flank-eGFP IAV infection at an MOI of 1.0 for various time points up to twenty-four hours. At the first-hour post-IAV infection, no significant difference in the levels of vRNAs including NP, NS1, eGFP, and M1 were detected between DMSO- and 526A-treated cells (Fig 2A, S4 and S5 Figs). During the early phase of IAV infection, incoming IAV genomes are the major source of vRNAs in infected cells. Thus our result suggested that DMSO- and 526A-treated cells had similar IAV infection rate and 526A did not block the entry of IAV. The levels of vRNAs increased dramatically at six hours post-infection indicating active replication of IAV. However, the expression of vRNAs was reduced at six hours post-infection in the 526A-treated A549-PB1 cells implies that 526A inhibited the replication of IAV (Fig 2A and S4 Fig). Similarly, the transcription of NP, NS1, eGFP, and M1 was significantly lower in the presence of 526A (Fig 2A and S4 Fig). To rule out the reduction of the transcription of viral mRNAs observed in 526A-treated A549-PB1 cells is due to the defect in host cell general transcription machinery or the RNA processing by 526A treatment, we quantified the expression levels of three housekeeping genes (SDHA, β-actin and 18S) in both the DMSO-treated and 526A-treated samples (S6 Fig). We observed no significant difference in the expression level of all three housekeeping genes, suggesting that 526A does not inhibit the host cell general transcription machinery or RNA processing.

To investigate whether the influenza protein level was affected by 526A, we performed immunoblotting on whole cell extracts with an antibody against NP protein. The NP protein was readily detected in DMSO-treated cells twelve hours post-IAV infection (Fig 2B and S7 Fig) but was reduced significantly in 526A-treated A549-PB1 cells. Our finding indicated that 526A reduces viral protein synthesis.

To determine whether 526A inhibits viral replication, we measured the viral titer in the culture media collected from PR8-PB1flank-eGFP virus-infected A549-PB1 cells pre-treated with or without 526A. We quantified the infected cells with FACS by sorting the viral-infected, eGFP-positive cells (Fig 2C). Consistent with the microscopy analysis (Fig 1C), 526A-treated A549-PB1 cells showed a 24% reduction of total eGFP-positive cells compared to DMSO-treated control A549-PB1 cells (Fig 2C, 2D and 2E left panel and S8 Fig). Among the eGFP-positive cells, there were about 5%% of 526A-treated cells showed high expression level of eGFP while about 40%% high eGFP-expressing cells were observed in the DMSO-treated sample (Fig 2C and S8 Fig). Importantly, there was an 18 fold reduction of new virus generated in the presence of 526A (Fig 2E, right panel). Together, these results imply that 526A inhibits IAV replication.

### 526A represses IAV-induced interferon stimulating genes (ISGs) and cytokine expression

**Fig 3:**
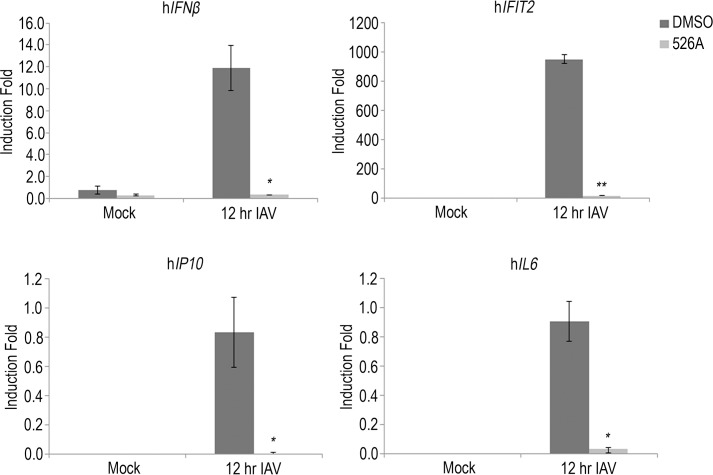
526A represses IAV-induced interferon stimulating genes (ISGs) and cytokine expression. A549-PB1 cells were pre-treated with or without 75 μM 526A for four hours and subjected to PR8-PB1flank-eGFP IAV infection at an MOI of 1.0 for twelve hours. The expression of *IFNβ*, *IFIT2*, *IP10* and *IL6* was measured with RT-qPCR (Table 1). Error bars represent the variation range of duplicate experiments. Student’s t-test: *, p < 0.05; **, p < 0.01.

In response to viral infection, the host cells will mount a type I interferon response to produce cytokines to eliminate the viral infection. To determine whether 526A suppresses IAV infection by amplifying the host cell antiviral responses, we quantitated the expression of ISGs and cytokines in IAV-infected A549-PB1 cells pre-treated with or without 75 μM of 526A. We infected A549-PB1 cells with PR8-PB1flank-eGFP IAV at an MOI of 1.0 for twelve hours and measured the gene expression with RT-qPCR (Table 1). All of the ISGs and cytokine genes, including *IFNβ*, *IFIT2*, *hIL6*, and *IP10* were induced upon PR8-PB1flank-eGFP IAV infection in DMSO-treated A549-PB1 cells (Fig 3 and S9 Fig). On the other hand, viral-induced the expression of *IFNβ*, *IFIT2*, *hIL6* and *IP10* were greatly reduced in 526A-treated A549-PB1 cells (Fig 3 and S9 Fig). These results indicate that 526A does not protect the host cells from IAV infection by enhancing the antiviral response.

**Table 1:** List of primers used in RT-qPCR assays.

Genes	Primers Sequence 5’–3’
*L32* Forward	AGCTCCCAAAAATAGACGCAC
*L32* Reverse	TTCATAGCAGTAGGCACAAAGG
*eGFP* Forward	AGATCCGCCACAACATCGAG
*eGFP* Reverse	TCTCGTTGGGGTCTTTGCTC
*M1* Forward	AAGACCAATCCTGTCACCTCTGA
*M1* Reverse	CAAAGCGTCTACGCTGCAGTCC
*NS1* Forward	CTTCGCCGAGATCAGAAATC
*NS1* Reverse	TGGACCATTCCCTTGACATT
*PR8-NP* Forward	ACGGCTGGTCTGACTCACAT
*PR8-NP* Reverse	TCCATTCCGGTGCGAACAAG
*SDHA* Forward	TGGGAACAAGAGGGCATCTG
*SDHA* Reverse	CCACCACTGCATCAAATTCATG
*β-actin* Forward	CGTCTTCCCCTCCATCG
*β-actin* Reverse	CTCGTTAATGTCACGCAC
*18S* Forward	GTAACCCGTTGAACCCCATT
*18S* Reverse	CCATCCAATCGGTAGTAGCG
*IFNβ* Forward	ACTGCCTCAAGGACAGGATG
*IFNβ* Reverse	AGCCAGGAGGTTCTCAACAA
*IFIT2* Forward	GCGTGAAGAAGGTGAAGAGG
*IFIT2* Reverse	GCAGGTAGGCATTGTTTGGT
*IP10* Forward	GACCAATGATGGTCACCAAA
*IP10* Reverse	GCAGGGTCAGAACATCCACT
*IL6* Forward	TACCCCCAGGAGAAGATTCC
*IL6* Reverse	TTTTCTGCCAGTGCCTCTTT
*TNFα* Forward	GCCCAGGCAGTCAGATCATCT
*TNFα* Reverse	TTGAGGGTTTGCTACAACATGG
PAMer Cy3	CCTCGCCAAGGGCCATCCTGTGCGCCA-CY

### 526A inhibits IAV-induced IRF3 and STAT1 activation

**Fig 4:**
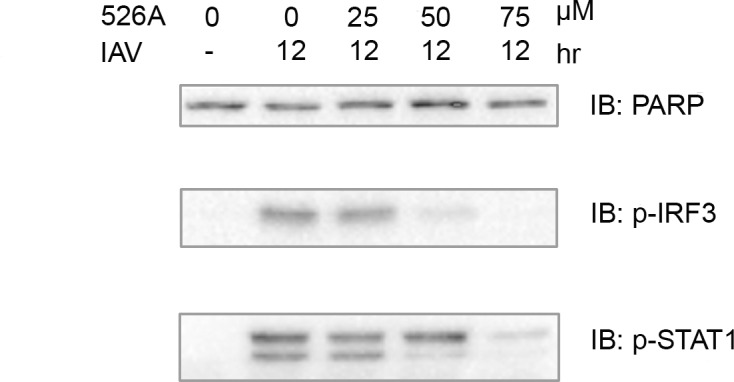
526A inhibits IAV-induced IRF3 and STAT1 activation. The A549-PB1 cells were treated with various concentrations of 526A as indicated for four hours followed by PR8-PB1flank-eGFP IAV infection at an MOI of 1.0 for twelve hours. Whole cell extracts were prepared and immunoblotted with antibodies against PARP, p-IRF3 and p-STAT1.

IRF3 pathway plays a crucial role in regulating the production of virus-induced ISGs and cytokines. Following RNA virus infection, IRF3 is activated, which, in turn, rapidly induce and activates the IFN-mediated transcription factor complex ISGF3, consisting of STAT1, STAT2 and IRF9 to produce ISGs and cytokines[12]. To determine whether 526A inhibits IAV-induced IRF3 and STAT1 activation, we measured the phosphorylation of IRF3 (p-IRF3) and STAT1 (p-STAT1), a biochemical hallmark of IRF3 and STAT1 activation respectively, in post viral-infected A549-PB1 cells with and without 526A treatment. We showed that 526A inhibits the activation of IRF3 in a dosage-dependent manner (Fig 4, middle panel and S10 Fig). IAV-induced phosphorylation of IRF3 was abolished when the A549-PB1 cells were pre-treated with 75 μM of 526A. Similarly, IAV-induced phosphorylation of STAT1 was decreased in the presence of 526A (Fig 4, bottom panel and S10 Fig). These results indicate that 526A inhibits IAV-induced activation of IRF3 and STAT1.

### 526A affects detection of viral RNA by RIG-I pathway

**Fig 5:**
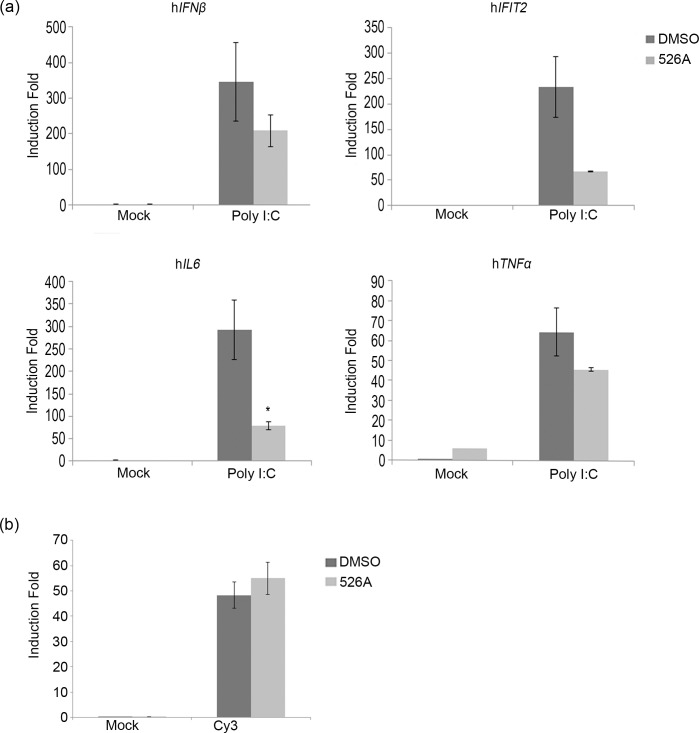
526A affects detection of viral RNA by RIG-I pathway. (a) A549-PB1 cells were pre-treated with or without 75 μM 526A for four hours. One hundred nanograms of poly I:C was transfected into the A549-PB1 cells using X-tremeGENE transfection method or added directly into the culture media without transfection (mock) for six hours. The expression of *IFNβ*, *IFIT2*, *IL6* and *TNFα* were measured with RT-qPCR. (b) A549-PB1 cells were pre-treated with or without 75 μM 526A for four hours. Twenty micromolar of PAMer-Cy3 was transfected into the A549-PB1 cells for twelve hours using X-tremeGENE transfection method. The signal of PAMer-Cy3 was measured using FACS. Error bars represent the variation range of duplicate experiments. Student’s t-test: *, p < 0.05.

Retinoic-acid-inducible protein 1 (RIG-I) is a key sensor of RNA virus infection (including *orthomyxovirus*) and activates antiviral responses via induction of interferons[13, 14]. Our results showed that 526A blocked the viral replication (Fig 2 and S4 Fig), virus-induced activation of IRF3 and STAT1 (Fig 4 and S10 Fig), and thus the IAV-induced induction of ISGs and cytokines (Fig 3 and S9 Fig). To test if 526A inhibits IAV-induced ISGs and cytokines production by blocking the RIG-I signaling pathway or simply through inhibiting the replication of IAV, we measured the expression of ISGs and cytokines in A549-PB1 cells transfected with polyinosinic-polyctidylic acid (poly I:C), which is a synthetic analog of double-stranded RNA (dsRNA), in the presence or absence of 526A. We observed that transfecting DMSO-treated A549-PB1 cells with poly I:C induced the expression of *IFNβ*, *IFIT2*, *IL6* and *TNFα*, while no expression was detected if poly I:C was added directly to culture media (Fig 5A, Table 1 and S11 Fig). Interestingly, 526A moderately inhibited poly I:C-induced expression of *IFNβ* and *TNFα*, and inhibited the expression of *IFIT2* and *IL6* by 70–80%% (Fig 5A and S11 Fig). To rule out the possibility that 526A affects the transfection efficiency, we also mimicked the experiment by transfecting PAMer-Cy3 in A549-PB1 cells with and without 526A and measured the PAMer-Cy3 positive cells with FACs (Fig 5B). The percentage of PAMer-Cy3 positive cells was similar between DMSO- and 526A-treated A549-PB1 cells (Fig 5B and S11 Fig). These results suggest that 526A treatment alone might partially block the RIG-I pathway.

## Materials and Methods

### Cell culture

A549-PB1 cells and HEK293T-PB1 cells were cultured in DMEM supplemented with 10%% fetal bovine serum (FBS), penicillin G (100 U/ml), and streptomycin (100 μg/ml).

#### Antibodies and compounds

Antibodies against actin (Santa Cruz Biotech), Hsp90α (Santa Cruz Biotech), p-IRF3 (Cell Signaling), p-STAT1 (Santa Cruz Biotech), PARP-1 (Santa Cruz Biotech) and NP (H16-L10-4R5, ATCC) were purchased from the respective commercial sources. 525A, 526A, 527A and 528A were dissolved in DMSO and DMSO was used as a mock treatment.

### Cell proliferation assay

A549-PB1 cells were treated with and without the four compounds respectively at various concentrations in a 96-well plate and incubated for two days at 37°C. Ten microlitres of 1 mg/ml 3-(4, 5-dimethylthiazol-2-yl)-2, 5-diphenyltetrazolium bromide (MTT) was added to each well followed by 4 hours incubation at 37°C in a CO_2_ incubator. The medium was carefully discarded. Then, 100 μl of DMSO was added to each well. After gently mixed, the absorbance was measured with a M200 PRO microplate reader (Tecan) at 540 nm wavelength. IC_50_ was calculated with GraphPad Prism 6 (Fig 1 and S1 Fig).

### Viral amplification

PR8 strain IAV carrying *enhanced green fluorescent protein (*eGFP) in place of the PB1 gene (PR8-PB1flank-eGFP virus) was amplified by infecting human embryonic kidney 293T cells that stably express PB1 protein (HEK239T-PB1 cells). Prior to virus infection, HEK293T-PB1 cells were washed once with PBS and then changed into influenza growth media (RPMI supplemented with 0.2%% bovine serum albumin, 0.01%% heat-inactivated fetal bovine serum, 100 U/ml penicillin, 100 μg/ml streptomycin and 1 mM calcium chloride) containing 4 μg/ml TPCK-treated trypsin. After 24 hours, the supernatant was harvested through a 0.45 μm filter. The virus titer was determined by FACS.

### Compound screening for antiviral activity

For screening against IAV, ten thousand A549-PB1 cells per well were seeded in a 96-well flat plate. After overnight incubation, cells were washed once with PBS and then changed into influenza growth media containing 0.2 μg/ml TPCK-treated trypsin. Fifty micromolar of four compounds were added respectively and incubated for four hours at 37°C. PR8-PB1flank-eGFP virus was then added at an MOI of 1.0. After eighteen hours, the eGFP of the cells was analyzed with an Olympus IX73 inverted microscope at 200X final magnification and photographed using an Olympus DP73 digital camera and Cellsens standard software (Fig 1C and S2 Fig).

For screening against VSV, ten thousand A549-PB1 cells per well were seeded in a 96-well flat plate. After overnight incubation, cells were washed once with PBS and then changed into influenza growth media containing 0.2 μg/ml TPCK-treated trypsin. Fifty micromolar of four compounds were added respectively and incubated for four hours at 37°C. Vesicular stomatitis virus carrying a GFP (VSV-GFP) was added at an MOI of 1.0 for sixteen hours (S3 Fig).

### Virus titering

Two hundred thousand of A549-PB1 cells were seeded in a 12-well plate with and without 75 μM of 526A for four hours followed by infecting cells with PR8-PB1flank-eGFP virus at an MOI of 0.75 in influenza growth media which contains 4 μg/ml TPCK-treated trypsin. Eighteen hours later, the supernatants were collected and filtered through 0.45 μm filters. The viruses in the collected supernatants were titered by infecting fresh A549-PB1 cells in influenza infection media containing 0.2 μg/ml TPCK-treated trypsin. After 24 hours, cells were washed with PBS and fixed with 0.1%% formaldehyde to inactivate the virus. Fixed cells were analyzed using a MACSQuant Analyzer (Miltenyi Biotec) to quantify eGFP-positive cells. Data were further analyzed with FlowJo software. Total infectious virus particle (IP) was calculated based on the following formula: IP/ml = (%% of the eGFP-positive cell) X (total cell number) / (total volume of supernatant used to infect the cells, in ml). One IP per cell was used when infecting cells at the MOI of 1.0.

### Immunoblotting

Two hundred thousand of A549-PB1 cells were seeded in a 12-well plate. After overnight incubation, culture media were removed and changed into IAV infection media and pre-treated with and without 526A at various concentrations for four hours. PR8-PB1flank-eGFP virus was added at an MOI of 1.0 for twelve hours. Whole cell lysate extracts were prepared from treated cells using a kinase lysis buffer (20 mM Tris pH 7.5, 120 mM sodium chloride (NaCl), 10%% glycerol, 1%% Triton X-100, 25 mM β-glycerophosphate, 1 mM sodium orthovanadate, 1 mM Dithiothreitol (DTT), and 1 mM phenylmethanesulfonylfluoride (PMSF)). After 30 minutes of on ice incubation, lysates were collected by centrifugation at 15 000 rpm 4°C for 10 minutes. Immunoblotting was performed using anti-NP, anti-actin, anti p-IRF3, anti p-STAT1 and anti-HSP90α antibodies. Western blot images were captured using ChemiDoc^TM^ MP System (Bio-Rad) (Figs 2B and 4). Images were processed using BioRad Image Lab version 5.0 and Adobe Photoshop CS4.

### Quantitative polymerase chain reaction (RT-qPCR)

Two hundred thousand of A549-PB1 cells were seeded in a 12-well plate. After overnight incubation, culture media were removed and changed into IAV infection media and pre-treated with and without 75 μM 526A for four hours. PR8-PB1flank-eGFP virus was added at an MOI of 1.0 for twelve hours. Total RNAs were isolated with the Thermo Scientific GeneJET RNA Purification Kit and complementary DNAs were synthesized with 2X SYBR Green PCR Master mix (Thermo Scientific). The checking of Quantitative PCR on the expression of IAV vRNAs, mRNAs, the expression of Interferon Stimulating genes and cytokines (Table 1) was run on a Bio-Rad Connect Real-Time PCR System. All data were normalized to *L32* (S4 Fig and S9 Fig).

### Cell transfection

For poly I:C transfection, four hundred thousand of A549-PB1 cells were seeded in a 12-well plate using Opti-MEM® 1X (Thermo Scientific) and pre-treated with or without 75 μM 526A. After four hours, 100 ng of Poly I:C (Tocris Bioscience) was transfected into the A549-PB1 cells using X-tremeGENE HP DNA transfection (Roche). After six hours, total RNAs were isolated and complementary DNAs were synthesized. Quantitative PCR was performed using Bio-Rad Connect Real-Time PCR System to measure the expression of Interferon Stimulating genes. All data were normalized to *L32* (Fig 5A and S11 Fig).

For PAMer-Cy3 transfection, four hundred thousand of A549-PB1 cells were seeded in a 12-well plate using Opti-MEM® 1X (Thermo Scientific) and pre-treated with or without 75 μM 526A. After four hours, 20 μM of Pamer-Cy3 was transfected into the A549-PB1 cells using X-tremeGENE HP DNA transfection (Roche). After twelve hours, cells were washed with PBS and fixed with 0.1%% formaldehyde. Fixed cells were analyzed using a MACSQuant Analyzer (Miltenyi Biotec) to quantify Pamer-Cy3 positive cells (Fig 5B and S11 Fig). Data were further analyzed with FlowJo software.

### Statistical analysis

Data were analysed with Microsoft Excel and presented as mean ± SD. Data are representative of two independent experiments. Statistical significance was assessed using two-tailed Student’s t-test.

## Discussion

Among all pathogens, RNA viruses impose a particular challenge due to their ability to evolve rapidly. Hence, there is an urgent need to develop new antiviral drugs against RNA viruses. In this study, we found that the four compounds, 525A, 526A, 527A and 528A, possess a different degree of antiviral activity against IAVs and VSVs in which 526A was selected for further study due to its potent antiviral property (Figs 1C and S3). We demonstrated that 526A suppresses the transcription of IAV vRNAs and the expression of IAV nucleoprotein (Fig 2 and S4 Fig). In addition, 526A blocks the IAV-induced expression of ISGs and cytokines (Fig 3 and S9 Fig). Moreover, 526A also represses the virus-induced activation of IRF3 and STAT1 (Fig 4 and S10 Fig). Furthermore, we found that 526A alone partially blocks the activation of RIG-I signaling pathway (Fig 5A and S11 Fig).

IAV infection in a host cell can be interfered via multiple ways including by inhibiting the entry of IAV, the release of vRNA, the entry of vRNA into the nucleus, restricting the viral genome replication or by blocking the budding of the newly synthesized virus [1]. Upon viral infection, type I interferon is activated to restrict initial viral replication before mounting the humoral immunity system[12]. Both toll-like receptors (TLRs) and the cytosolic RIG-I-like receptors (RLRs) are the first lines of defence that have been implicated in the induction of IFNs by influenza viruses[15]. Since TLR7/8 is absence in the A549-PB1 cells used in this study[16], therefore detection of IAV would be mediated primarily through the RIG-I pathway which recognizes viral 5’ppp or 5’pp RNA in the cytosol[15, 17, 18]. Our results show that 526A protects the host cells from IAV infection but unexpectedly, not through enhancing the type I interferon response in the host cells. In addition, we showed that similar amount of vRNAs were detected regardless in the presence or absence of 526A at the early phase of IAV infection (Fig 2A), while vRNAs or their transcripts were significantly lower in the presence of 526A at the later stages of IAV infection. These findings suggested that 526A blocks the replication of IAV at a step after the entry of IAV. Our data also show that the host cell general transcription machinery and RNA processing is unaffected by 526A treatment (S6 Fig). However, the fact that 526A inhibits the ISGs expression upon poly I:C treatment (Fig 5A), albeit with a lesser degree compare to IAV-induced expression of ISGs (Fig 3), further suggests that 526A may also directly inhibit the activation of RIG-I pathway. Further experiments are required to pinpoint the molecular mechanism of 526A-mediated inhibition of IAV replication.

## Supporting Information

S1 Fig Primary data of the MTT assay. (XML)

S2 Fig Bright field microscopy images of anti-IAV screening. (PDF)

S3 Fig Screening of four indole derivatives on VSV-GFP. (PDF)

S4 Fig Primary RT-qPCR data of 526A suppresses IAV replication. (XLSX)

S5 Fig 526A suppresses IAV replication (Y-axis transformed into Log_10_) (TIF)

S6 Fig Expression level of other housekeeping genes. A549-PB1 cells were pre-treated with or without 75 μM 526A for sixteen hours and the expression of three housekeeping genes (SDHA, β-actin and 18S) was measured with RT-qPCR. Error bars represent the variation range of duplicate experiments. Student’s t-test: *, p < 0.05; **, p < 0.01.
(TIF)

S7 Fig Full length blots of 526A inhibits NP. (PDF)

S8 Fig Primary data of virus titering. (XLSX)

S9 Fig Primary data of 526A represses IAV-induced Interferon Stimulating Genes and Cytokines Expression. (XLSX)

S10 Fig Full length blots for 526A suppresses p-IRF3 and p-STAT1. (PDF)

S11 Fig Primary data of 526A affects detection of viral RNA by Rig-I pathway. (XLSX)
