# Supplementary figures and images for: Identification of 5-Methoxy-2-(Diformylmethylidene)-3,3-Dimethylindole as an Anti-Influenza A Virus Agent
Source: PLoS One. 2017 Jan 23;12(1):e0170352. doi: 10.1371/journal.pone.0170352 (PMC5256998; doi:10.1371/journal.pone.0170352)

DMSO

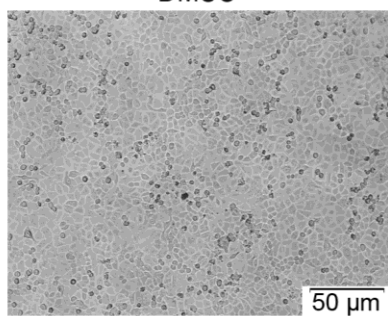

50  $\mu\text{M}$  525A

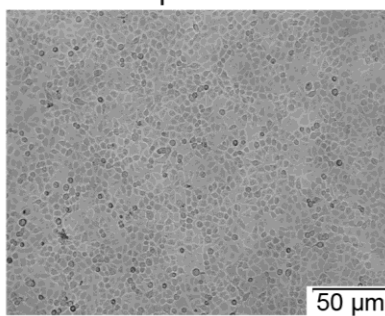

50  $\mu\text{M}$  526A

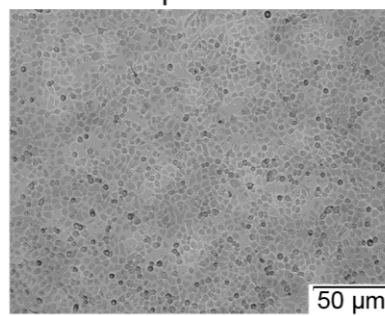

50  $\mu\text{M}$  527A

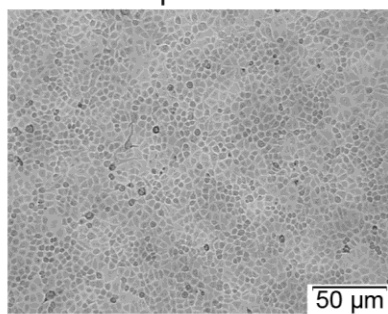

50  $\mu\text{M}$  528A

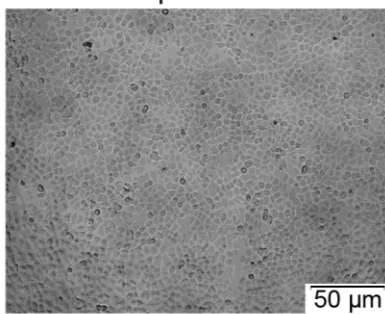

Supplement: S2 Fig — (PDF) [file pone.0170352.s002.pdf]

a)

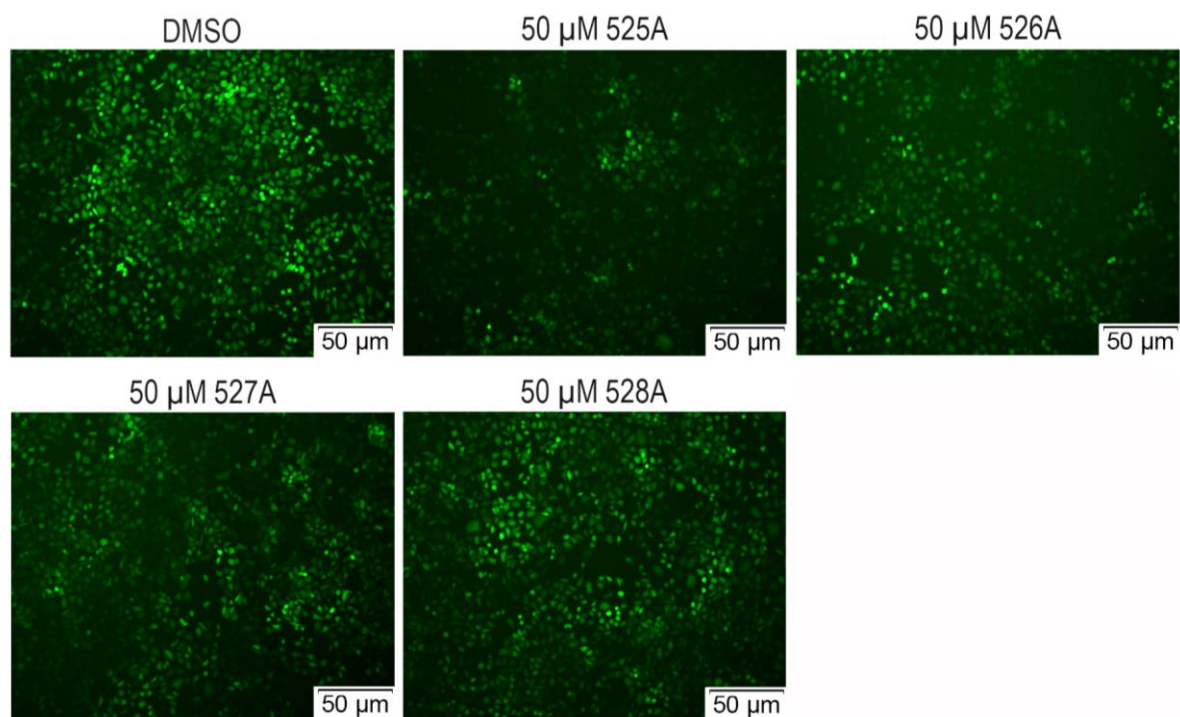

b)

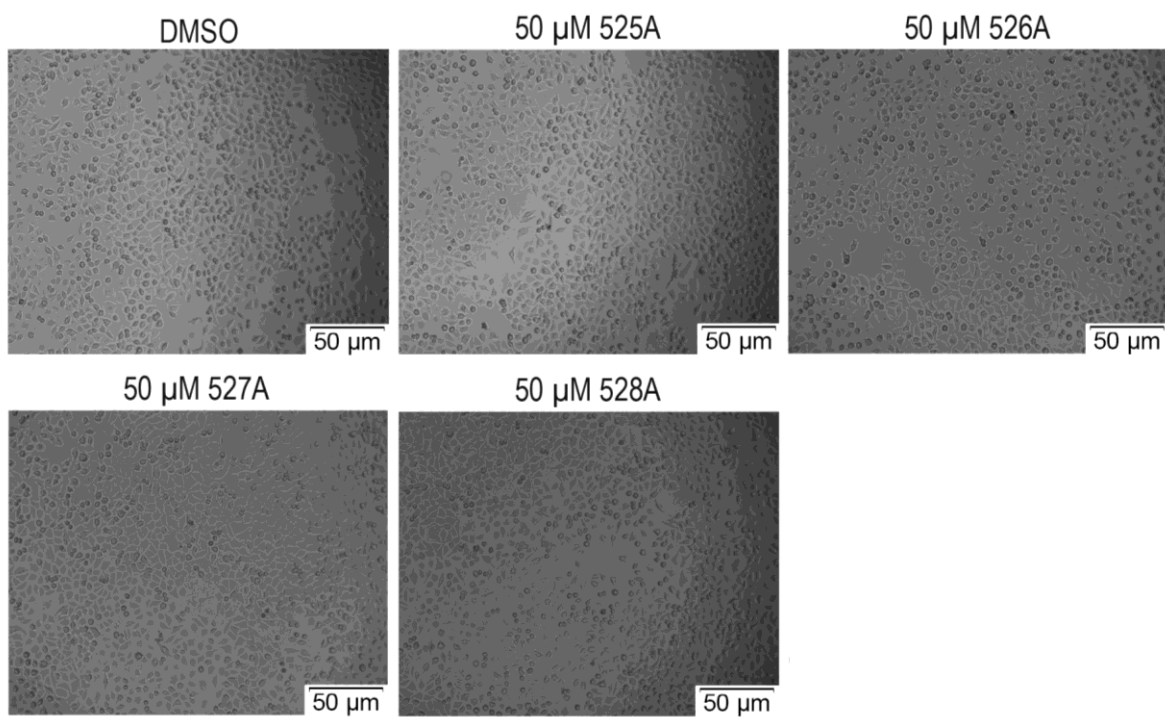

Supplement: S3 Fig — (PDF) [file pone.0170352.s003.pdf]

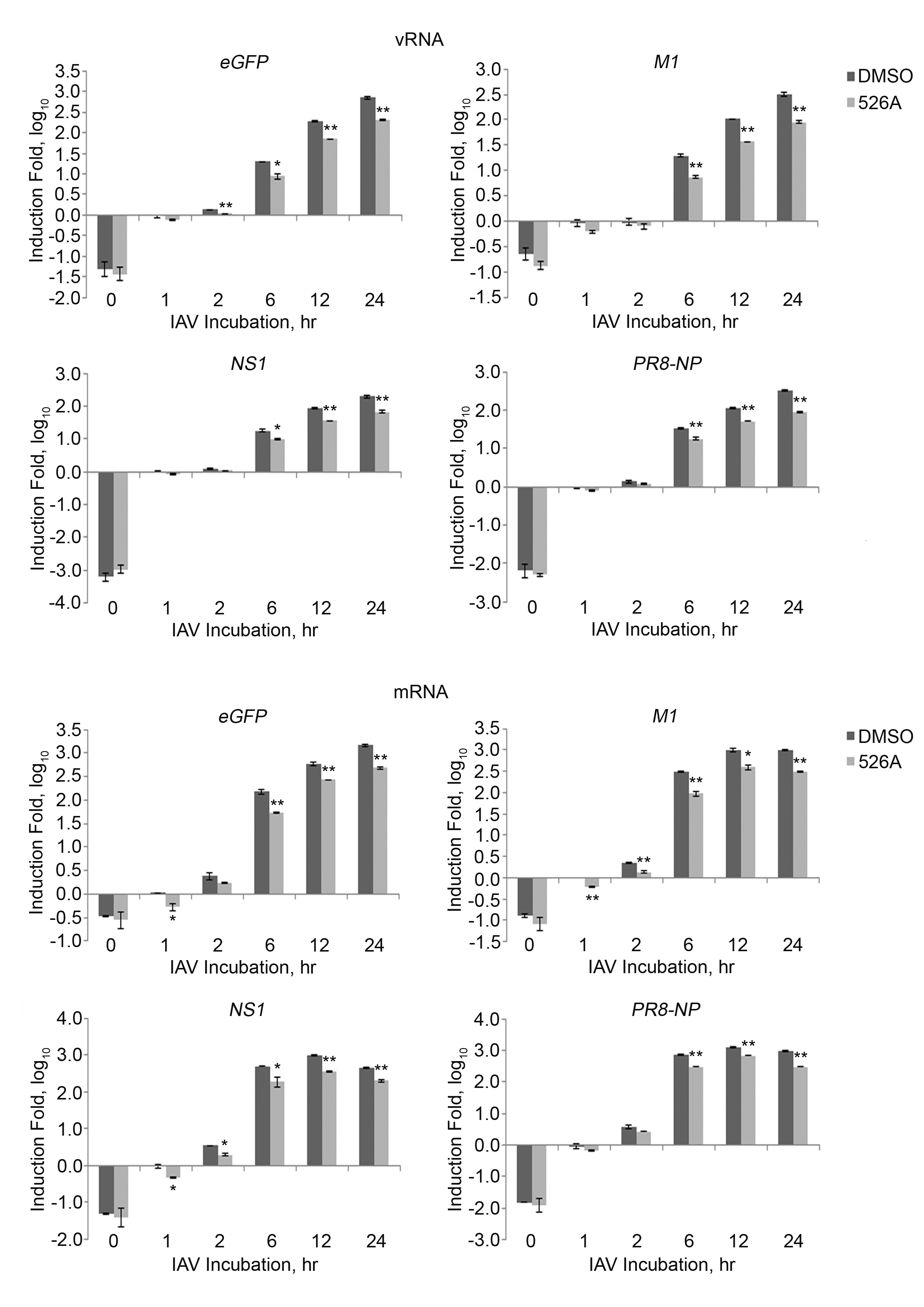

Supplement: S5 Fig — (TIF) [file pone.0170352.s005.tif]

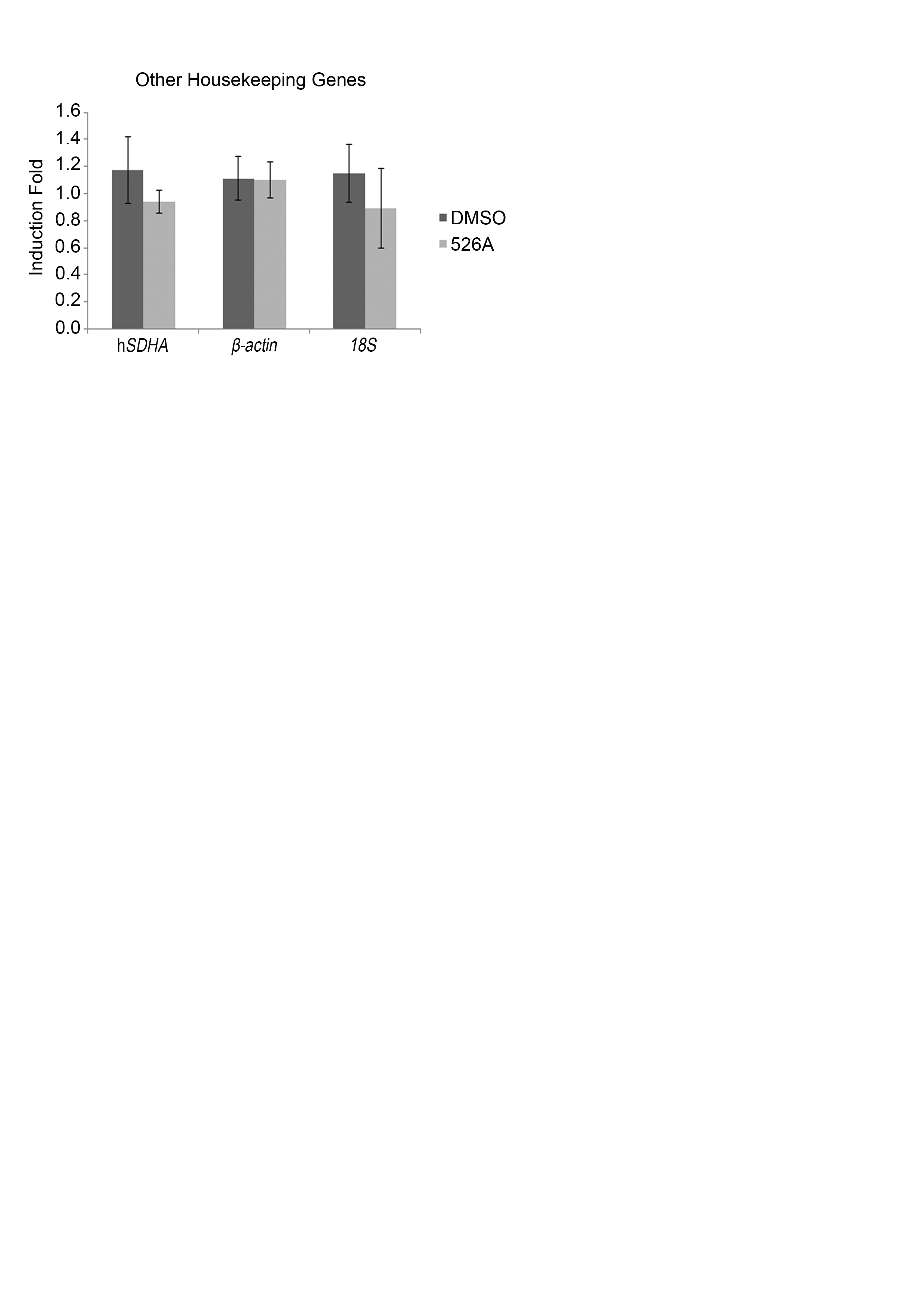

Supplement: S6 Fig — A549-PB1 cells were pre-treated with or without 75 μM 526A for sixteen hours and the expression of three housekeeping genes (SDHA, β-actin and 18S) was measured with RT-qPCR. Error bars represent the variation range of duplicate experiments. Student’s t-test: *, p < 0.05; **, p < 0.01. (TIF) [file pone.0170352.s006.tif]

|      |   |    |    |    |         |
|------|---|----|----|----|---------|
| 526A | 0 | 0  | 75 | 75 | $\mu$ M |
| IAV  | - | 12 | -  | 12 | hr      |

55 kDa -

40 kDa -

IB: NP

35 kDa -

25 kDa -

IB: Actin

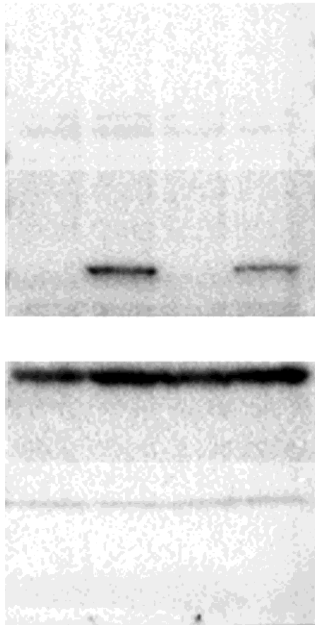

Supplement: S7 Fig — (PDF) [file pone.0170352.s007.pdf]

| 526A | 0 | 0  | 25 | 50 | 75 | $\mu$ M |
|------|---|----|----|----|----|---------|
| IAV  | - | 12 | 12 | 12 | 12 | hr      |

130 kDa -  
100 kDa -

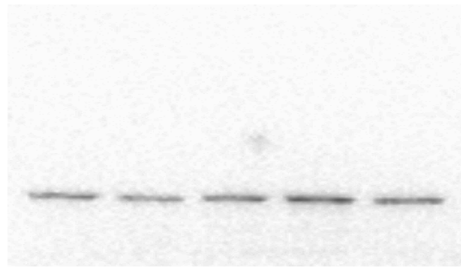

IB: PARP

55 kDa -  
40 kDa -

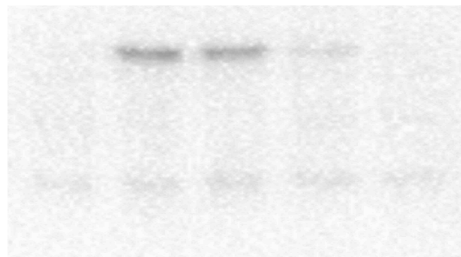

IB: p-IRF3

100 kDa -  
70 kDa -

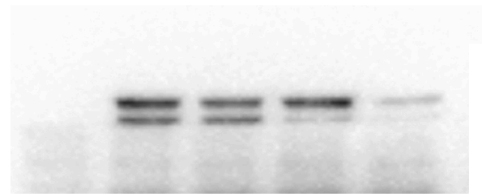

IB: p-STAT1

Supplement: S10 Fig — (PDF) [file pone.0170352.s010.pdf]
